# Multifaceted Human Antigen R (HuR): A Key Player in Liver Metabolism and MASLD

**DOI:** 10.3390/livers5030033

**Published:** 2025-07-21

**Authors:** Natalie Eppler, Elizabeth Jones, Forkan Ahamed, Yuxia Zhang

**Affiliations:** Department of Pharmacology, Toxicology and Therapeutics, University of Kansas Medical Center, MS 1018, 3901 Rainbow Boulevard, Kansas City, KS 66160, USA

**Keywords:** RNA binding proteins, human antigen R (HuR), metabolic dysfunction-associated steatotic liver disease (MASLD), metabolic syndrome, post-transcriptional regulation, non-alcoholic fatty liver disease (NAFLD), hepatic steatosis

## Abstract

Metabolic dysfunction-associated steatotic liver disease (MASLD) has become the leading cause of chronic liver disease worldwide, affecting approximately 25–30% of the global adult population and highlighting the urgent need for effective therapeutics and prevention strategies. MASLD is characterized by excessive hepatic lipid accumulation and can progress, in a subset of patients, to metabolic dysfunction-associated steatohepatitis (MASH), a pro-inflammatory and pro-fibrotic condition associated with increased risk of liver cirrhosis and hepatocellular carcinoma. Although the molecular drivers of MASLD progression remain incompletely understood, several key metabolic pathways—such as triglyceride handling, cholesterol catabolism, bile acid metabolism, mitochondrial function, and autophagy—are consistently dysregulated in MASLD livers. This narrative review summarizes primary literature and highlights insights from recent reviews on the multifaceted role of the mRNA-binding protein Human antigen R (HuR) in the post-transcriptional regulation of critical cellular processes, including nutrient metabolism, cell survival, and stress responses. Emerging evidence underscores HuR’s essential role in maintaining liver homeostasis, particularly under metabolic stress conditions characteristic of MASLD, with hepatocyte-specific *HuR* depletion associated with exacerbated disease severity. Moreover, comorbid conditions such as obesity, type 2 diabetes mellitus, and cardiovascular disease not only exacerbate MASLD progression but also involve HuR dysregulation in extrahepatic tissues, further contributing to liver dysfunction. A deeper understanding of HuR-regulated post-transcriptional networks across metabolic organs may enable the development of targeted therapies aimed at halting or reversing MASLD progression.

## 1. Introduction

Metabolic dysfunction-associated steatotic liver disease (MASLD) is now recognized as the most common cause of chronic liver disease, affecting approximately 25–30% of the global adult population [1,2]. A recent modeling study projected that the prevalence of MASLD in the United States will rise from 99.2 million in 2024 to 133.5 million by 2044, representing a 35% increase [3]. In addition to its clinical and epidemiological impact, MASLD imposes a substantial economic burden. In 2016 alone, direct medical costs attributable to MASLD in the United States exceeded $103 billion [4]. Under a no-treatment scenario, cumulative direct U.S. healthcare costs related to MASLD are projected to reach $520.3 billion between 2024 and 2044 [3]. As the societal and financial burden of MASLD continues to grow, the development of effective therapeutic and preventive strategies has become an urgent public health priority [5].

Although MASLD was formerly referred to as non-alcoholic fatty liver disease (NAFLD), the nomenclature has evolved to better reflect its metabolic etiology. In 2020, an international panel of experts proposed the term metabolic dysfunction-associated fatty liver disease (MAFLD), which incorporated cardiometabolic risk factors into its diagnostic criteria and removed alcohol consumption as a negative criterion [6]. Building on this, a 2023 consensus among liver societies and patient advocacy groups adopted the term MASLD as a more accurate and less stigmatizing descriptor [7]. MASLD is defined as excessive hepatic triglyceride accumulation (>5% liver weight) in the presence of at least one additional cardiometabolic risk factor and absence of moderate to excessive alcohol consumption [7]. The cardiometabolic risk factors include (1) Body mass index (BMI) ≥ 25 kg/m^2^ or waist circumference >94 cm in men and >80 cm in women, with ethnicity taken into account, (2) Fasting blood glucose ≥ 100 mg/dl or 2 hrs post-load glucose level ≥ 140 mg/dl or hemoglobin A1C (HbA1c) ≥ 5.7%, (3) Blood pressure ≥ 130/85 mmHg, (4) Plasma triglycerides ≥ 150 mg/dl, and 5) Plasma high-density lipoprotein (HDL) cholesterol <40 mg/dl for men or <50 mg/dl for women [7]. An additional category, termed metabolic alcohol related/associated liver disease (MetALD), was chosen to define metabolic liver disease in the presence of moderate alcohol consumption [7]. Importantly, more than 96% of individuals who originally met NAFLD-diagnostic criteria also meet MASLD-diagnostic criteria, allowing for continuity between NAFLD-related and MASLD-related studies [8].

MASLD progression from simple, non-inflammatory metabolic dysfunction-associated steatotic liver (MASL) to pro-inflammatory and pro-fibrotic metabolic dysfunction-associated steatohepatitis (MASH) is associated with poor outcomes and limited treatment success [9]. In 10–30% of MASLD patients, progression to MASH and MASH with fibrosis significantly increases the risk of liver cirrhosis, hepatocellular carcinoma (HCC), cardiovascular events, and extrahepatic malignancies [1,10,11]. Although lifestyle modifications, including dietary changes and increased physical activity, can reverse MASLD, long-term efficacy is often hindered by poor adherence [12]. To address this need, resmetirom, a liver-specific thyroid hormone receptor β agonist, became the first and only Food and Drug Administration (FDA)-approved pharmacotherapy for MASH in 2024 [13]. In parallel, recent phase 2 and 3 clinical trials have demonstrated the therapeutic potential of glucagon-like peptide-1 (GLP-1) receptor agonists (e.g., liraglutide, semaglutide), GIP/GLP-1 dual receptor agonists (e.g., tirzepatide), glucagon/GLP-1 dual receptor agonists (e.g., pemvidutide, survodutide), and glucagon/GIP/GLP-1 triple receptor agonists (e.g., efocipegtrutide) for the treatment of MASH [14–18]. While the phase 2 trial of efocipegtrutide is ongoing with estimated completion in November 2025, results from other trials support the promise of GLP-1-based therapies—alone or in combination—as effective pharmacologic options for MASLD/MASH [19]. Nevertheless, gastrointestinal side effects remain a major limitation, and larger, long-term studies are needed to fully evaluate their safety and efficacy [20]. Continued efforts to identify MASLD-targeted therapies are therefore essential, and elucidating the molecular mechanisms that drive progression from MASLD to MASH remains a critical research priority. Moreover, MASLD frequently coexists with other metabolic disorders—such as obesity, cardiovascular disease (CVD), and type 2 diabetes mellitus (T2DM)—which can exacerbate liver pathology and further underscore the need to investigate inter-organ metabolic crosstalk [21].

Human antigen R (HuR), encoded by the embryonic lethal abnormal vision-like 1 (*Elavl1*) gene [22], is a member of the Hu family of RNA-binding proteins (RBPs) and functions as a dynamic regulator of mRNA processing and stability, exerting context-dependent roles across various cellular processes and disease states. The *Elavl* gene family was first identified in *Drosophila* as essential for embryonic and ocular development [23]. In humans, Hu proteins selectively bind adenine- and uridine-rich elements (AREs) within the 3′ untranslated regions (3′ UTRs) of mature mRNAs and in intronic sequences of pre-mRNAs [24]. AREs are present in approximately 22% of human 3′ UTRs and 25% of introns [25], and are typically found in transcripts encoding proteins involved in development, inflammation, metabolism, and signal transduction. While Hu family members HuB, HuC, and HuD are predominantly expressed in the nervous system, HuR is ubiquitously expressed and essential for normal development and homeostasis [26]. HuR was originally appreciated for its role in stabilizing mRNAs within the early-response gene family, including Fos proto-oncogene (*c-fos*), *c-myc*, and interleukin-3 (*Il3*) [22,27]. Overtime, however, HuR has since emerged as a dynamic, context-dependent modulator of numerous biological processes, including cell cycle progression [28–30], differentiation [31,32], apoptosis [33,34], and senescence [35,36] through regulation of various transcripts, such as Cyclin D1 (*CCND1*) [37], transforming growth factor β (*TGFB*) [38], and cyclin dependent kinase inhibitor 1A (*CDKN1A*, p21) [39]. Various factors influence HuR activity, including cell type, subcellular localization, and environmental cues. For example, cytoplasmic localization of HuR in liver-resident hepatic stellate cells (HSCs) promotes liver fibrosis development [40,41], while inhibition of HuR in adipocytes is associated with obesity and insulin resistance [42,43]. HuR’s regulatory role is variable and diverse, providing ample opportunity for continued study of HuR’s functions in tissue-specific homeostasis and disease progression.

The rationale for this narrative review is based on HuR’s emerging role as a critical post-transcriptional regulator of metabolic homeostasis, inflammation, and fibrosis—the hallmark features of MASLD and its progression to MASH. Given HuR’s cell-type–specific and stimulus-responsive activity, its function in different tissues may either promote or protect against MASLD depending on the biological context. This review provides a comprehensive synthesis of current evidence on HuR’s role in liver pathophysiology, focusing on its regulation of lipid metabolism, bile acid synthesis, mitochondrial function, oxidative stress, cell death and survival, autophagy, inflammation, and fibrogenesis. Due to the recent shift in metabolic liver disease nomenclature to include cardiometabolic risk factors as diagnostic criteria, we also highlight HuR’s involvement in extrahepatic metabolic tissues, including adipose, cardiac, and immune cells, where its dysregulation contributes to comorbid conditions such as obesity, CVD, and T2DM, all of which exacerbate MASLD. Finally, we discuss both the potential benefits and challenges of targeting HuR in MASLD and related metabolic disorders, emphasizing the need for cell-specific strategies that preserve HuR’s protective roles while mitigating its pathogenic effects.

## 2. Review Methodology

In this narrative review, we searched the PubMed database for literature related to the role of HuR in overall liver function and MASLD development, while also including references addressing metabolic comorbidities such as CVD, T2DM, and obesity. Except for adipose, cardiovascular, and immune tissues, studies focusing on HuR function in other extrahepatic tissues and disease contexts were excluded, as these topics have been extensively reviewed elsewhere and fall outside the scope of this review. References were not restricted by publication date; however, efforts were made to prioritize the most relevant and up-to-date sources pertaining to the themes discussed.

## 3. HuR Structure and Subcellular Localization

**Figure 1.**
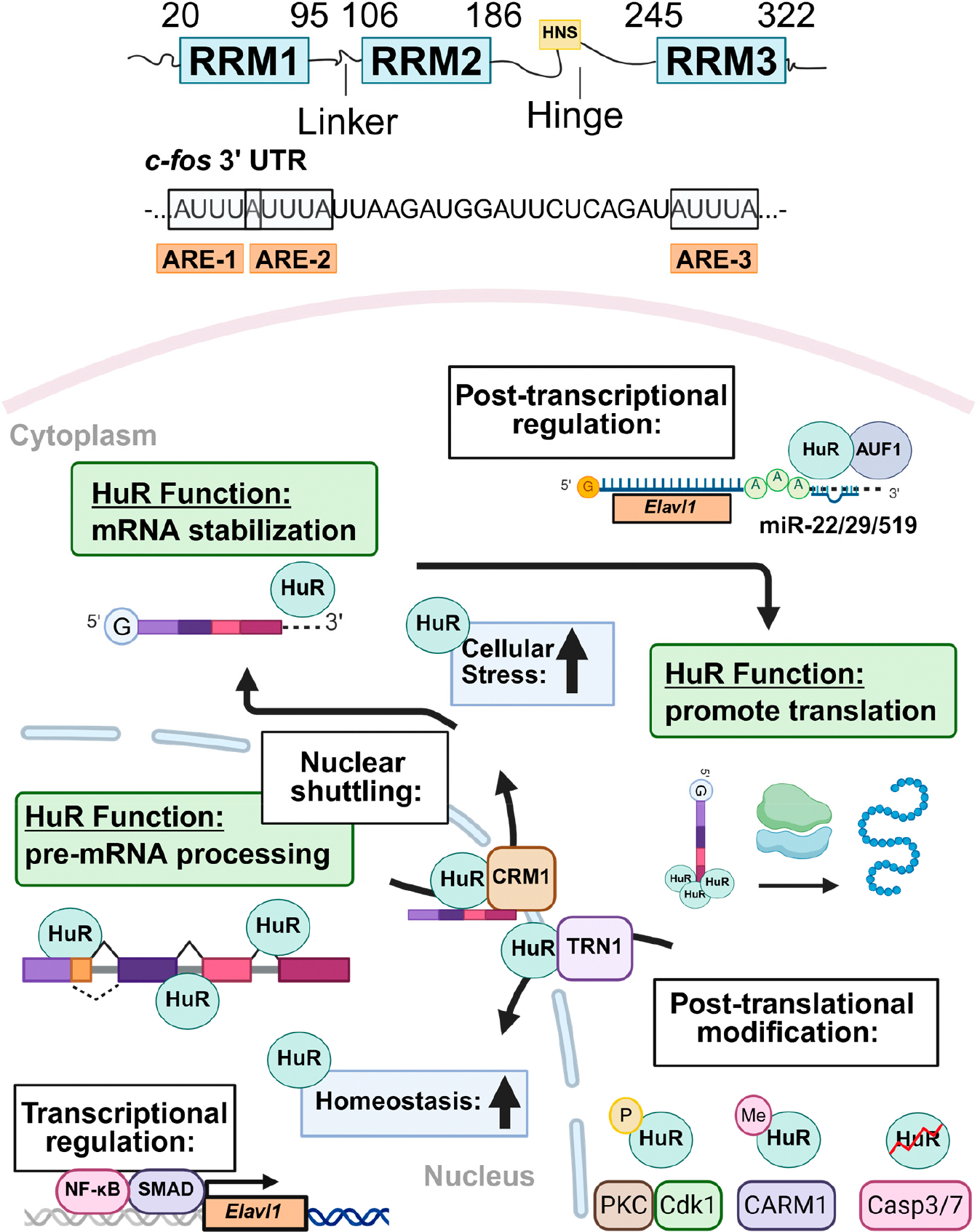
Schematic overview of HuR structure, function, and regulatory mechanisms. HuR (Human antigen R) contains three highly conserved RNA recognition motifs (RRMs) connected by flexible hinge and linker domains. The HuR nucleocytoplasmic shuttling sequence (HNS) enables HuR to move between the nucleus and cytoplasm. HuR binds directly to target mRNAs by recognizing adenine- and uridine-rich elements (AREs) through its RRMs, classically exemplified by its interaction with transcripts such as the Fos proto-oncogene (*c-fos*). The regulation of HuR expression and activity is multifactorial. Transcriptional regulators of HuR include nuclear factor kappa B (Nf-κB) and mothers against decapentaplegic homolog (SMAD). At the post-transcriptional level, HuR positively autoregulates its own expression, while ARE/poly(U)-binding degradation factor 1 (AUF1) and several miRNAs, including miR-22/29/519, negatively regulate HuR. Several post-translational modifications, including phosphorylation (P) by protein kinase C (PKC) and cyclin-dependent kinase 1 (Cdk1), methylation (Me) by coactivator-associated arginine methyltransferase 1 (CARM1), and protein cleavage mediated by cysteine- and aspartate-protease family members Casp3/7 (caspase 3/7). Protein–protein interactions collectively govern its localization and functional output. Under homeostatic conditions, HuR is predominantly localized in the nucleus, where it binds to introns and splice sites of pre-mRNAs to regulate pre-mRNA processing. In response to cellular stress, HuR protein translocates to the cytoplasm, where it enhances stability and translation of mature mRNA targets involved in stress response, survival, inflammation, and metabolism. Created in BioRender V2 https://BioRender.com/z74o1fi. (accessed on 1 July 2025).

HuR was first cloned and characterized by Ma et al., who used degenerate oligonucleotide-directed PCR to amplify a 140-nucleotide fragment from HeLa cells [22]. This gene product was called HuR and was highly similar, but distinct, in sequence to mRNAs encoding HuB, HuC, and HuD. The protein encoded by *HuR*/*Elavl1* comprises a short 20 amino acid long N-terminus that precedes three RNA recognition motifs (RRMs) (Figure 1). RRM1 and RRM2 are connected via a 12 amino acid linker domain, while a much longer 60 amino acid hinge domain connects RRM2 to RRM3 [22,44,45]. All three RNA RRMs are highly conserved among Hu protein family members and exhibit strong affinity and specificity for AREs, which are commonly located in the 3′ UTRs and intronic regions of many labile and stress-inducible mRNAs [26]. Structural studies of RNA-bound HuR reveal that binding of RRM2 to AREs is contingent upon conformational changes initiated by RRM1′s interaction with the RNA [44,45]. Although non-covalent interactions between RRM1/2 and AREs temporarily anchor HuR to target transcripts, full functional activity of HuR requires the involvement of RRM3. Specifically, RRM3 enhances HuR’s binding affinity by mediating interactions with the poly(A) tail [46] and promoting HuR dimerization [45], which together facilitate the cooperative binding of multiple HuR molecules to tandem AREs. Additionally, HuR binding to other proteins, including SET nuclear proto-oncogene isoform α (SETα), SET nuclear proto-oncogene isoform β (SETβ), and a proliferation-inducing ligand (APRIL), is also RRM3 dependent [47].

Multiple factors regulate the subcellular localization of HuR. Early studies demonstrated that HuR resides primarily in the nucleus of eukaryotic cells [48]. However, ARE-mediated mRNA decay is primarily cytosolic, raising concern regarding how HuR could perform its well-recognized mRNA stabilizing function if retained within the nucleus. To resolve this, Fan and Steitz employed heterokaryon fusion experiments, showing that Flag-tagged HuR shuttles between the nucleus and cytoplasm [48]. Later, HuR deletion mutants were used to identify a non-canonical sequence within the hinge domain of HuR that mediated both nuclear uptake and export of HuR, termed the HuR nucleocytoplasmic shuttling sequence (HNS) [49]. It is now established that HuR’s nucleocytoplasmic transport depends on interactions between the HNS and specific nuclear transport factors [50,51], including chromosomal region maintenance 1 (CRM1) [52], transportin 1 and 2 (TRN1/2) [53], APRIL [47], and importin-α (IMPα) [54]. This transport is further modulated by post-translational modifications (PTMs) of either HuR or its transport factors. For instance, in the human colorectal carcinoma RKO cell line, the activation of AMP-activated protein kinase (AMPK) leads to the phosphorylation of IMPα1 at Ser105 and acetylation at Lys22, enhancing its binding to HuR and promoting HuR nuclear import [54]. In HeLa cells, CDK1-mediated phosphorylation of HuR facilitates its binding to the adaptor protein 14–3-3, resulting in nuclear retention during the G2 phase of the cell cycle [55]. Additionally, *TRN2* knockdown in HeLa cells decreases nuclear retention and increases cytoplasmic accumulation of HuR, highlighting TRN2’s essential role in HuR nuclear import [56].

HuR export from the nucleus into the cytoplasm is primarily triggered by heightened cellular stress [57], increased cellular proliferation [58,59], and exposure to proinflammatory stimuli [60]. For example, in HeLa cells, heat shock promotes the cytoplasmic translocation of HuR through CRM1-mediated nuclear export, facilitated by protein–protein interactions between HuR and nuclear transport factors APRIL and phosphoprotein 32 (pp32) [52]. DNA damage promotes phosphorylation of HuR within the N-terminal region, leading to both increased cytoplasmic HuR localization and HuR oligomerization in HeLa cells [57]. During myoblast differentiation, a 24 kDa HuR cleavage product competitively inhibits the nuclear transport factor TRN2, leading to cytoplasmic accumulation of HuR [61].

HuR dysregulation, particularly its altered subcellular localization, has been extensively studied in disease contexts over the past 25 years. Increased cytoplasmic localization of HuR is widely recognized as a poor prognostic indicator in multiple cancer types [58]. Highlighting the critical role of cytoplasmic HuR in promoting tumorigenesis, small-molecule inhibitors that disrupt HuR nuclear–cytoplasmic shuttling have shown efficacy in reducing tumor growth in both in vitro and in vivo models [58]. In the context of liver diseases, cytoplasmic HuR staining has been correlated with more advanced stages of HCC [62], and phosphorylation of HuR by protein kinase C-δ (PKC-δ) has been shown to increase its cytoplasmic localization in Huh7.5 liver cancer cells during hepatitis C virus infection [63]. Furthermore, in a murine model of acute hepatic ischemia–reperfusion injury, HuR protein levels rise in the cytoplasm following reperfusion, and stimulation of primary murine hepatocytes with pro-inflammatory cytokines similarly promotes cytoplasmic HuR accumulation [60].

In summary, the nucleocytoplasmic shuttling of HuR depends on the interaction of its HNS sequence with nuclear transport factors. PTMs further modulate HuR’s subcellular localization. Notably, HuR localization dynamically shifts between physiological and pathological conditions, reflecting its context-dependent regulatory roles in various diseases.

## 4. Regulation of HuR Expression and Protein Stability

HuR expression is tightly regulated at multiple levels, including transcriptional regulation, post-transcriptional control by RNA-binding proteins and microRNAs (miRNAs), PTMs, and protein–protein interactions (Figure 1). Transcriptional regulation of HuR plays a critical role in the cellular stress response and tumorigenesis. In mice (NCBI Gene ID: 15568), four annotated HuR transcripts exist; however, all encode the same 326-amino acid protein. These transcript variants differ primarily in the sequence and secondary structure of their 3′ and 5′ UTR [64–66]. The *HuR* gene contains three putative transcriptional start sites located upstream of the methionine start codon. These sites are flanked by transcription factor binding motifs, including ETS proto-oncogene 1 (ETS1), cAMP responsive element binding protein 1 (CREB), and activator protein-1 (AP-1) elements. Several transcription factors have been shown to bind directly to the *HuR* promoter (Figure 1). For example, nuclear factor κB (NFκB) enhances transcription of the long HuR variant by direct binding of the NFκB p65 subunit to the promoter, promoting cell survival and proliferation in MKN74 gastric cancer cells [67]. Similarly, ATP depletion activates mothers against decapentaplegic homolog (SMAD)-mediated transcription of the short HuR variant in LLC-PK1 porcine kidney proximal tubule cells, also contributing to cell survival [68]. The presence and differential regulation of both short and long *HuR* transcript variants enable context-specific fine-tuning of HuR expression.

Beyond transcriptional control, *HuR* expression is modulated at the post-transcriptional level by RNA-binding proteins and miRNAs, which directly interact with *HuR* mRNA to influence its stability, translation, and subcellular localization [69,70] (Figure 1). In fact, HuR can positively autoregulate its own expression by binding to its cognate mRNA, enhancing its stability, cytoplasmic localization, and translation [36,69]. In contrast, the decay-promoting RNA-binding protein ARE/poly(U)-binding degradation factor 1 (AUF1) accelerates *HuR* degradation by directly binding to the *HuR* transcript and targeting it to exosomes for mRNA decay [69,71]. Direct binding of miRNAs, such as miR-22, miR-29, and miR-519, to the *HuR* 3′ UTR also represses *HuR* expression via multiple mechanisms [72–74]. For example, in multiple cancer cell lines, direct binding of miR-519 to both the 3′ UTR and the coding region of *HuR* represses translation but not the stability of *HuR* [74]. In addition, the tumorigenicity of the SW480 colorectal cancer cell line is suppressed by miR-22-mediated repression of HuR expression [72]. In the context of liver disease, miR-22 is downregulated during MASLD progression and HCC development [75], which may contribute to HuR upregulation in HCC.

Furthermore, HuR protein stability and RNA-binding capacity are intricately regulated by PTMs [76] and protein–protein interactions [45] (Figure 1). For a comprehensive overview of HuR PTMs, we refer readers to an excellent review [76]. Briefly, phosphorylation of HuR by kinases such as AMPK [54], CDK1 [55], checkpoint kinase 2 (CHK2) [77,78], mitogen-activated protein kinase p38 (MAPK p38) [39,79], PKC-α [80], and PKC-δ [63] regulates not only HuR’s nucleocytoplasmic shuttling but also its stability and RNA-binding affinity. Of clinical relevance, pharmacological activation of AMPK is considered a potential therapeutic strategy for MASLD [81], while AMPK activation also increases HuR nuclear localization [54]. In addition, methylation of HuR enhances the stability of its target mRNAs and promotes its cytoplasmic accumulation in cancer cells [35], whereas severe cellular stress induces caspase-mediated cleavage of cytoplasmic HuR, generating active fragments with distinct RNA-binding profiles and pro-apoptotic functions [33,34]. Furthermore, biophysical and biochemical analyses using techniques such as fluorescence resonance energy transfer (FRET), X-ray crystallography, and immunoprecipitation have revealed HuR engaging in non-covalent interactions with itself and other proteins to further promote expression of target transcripts [45,82,83]. Together, these multilayered regulatory mechanisms underscore the complexity of HuR regulation and function, enabling dynamic and context-specific control of its activity in response to diverse cellular signals. The diverse mechanisms governing HuR expression, localization, and function are summarized in Table 1.

## 5. Overview of HuR Function During Physiological and Pathological Conditions

### 5.1. Nuclear Functions of HuR Under Physiological Conditions

Under physiological conditions, HuR is predominantly localized to the nucleus, where it plays key roles in splicing, 3′ end processing, pre-mRNA stabilization, and nuclear export of target transcripts (Figure 1). For instance, approximately 35% of HuR binding sites identified in unstressed HeLa cells by photoactivatable ribonucleotide crosslinking and immunoprecipitation were located within intronic regions, suggesting a major role in pre-mRNA processing [84]. Consistent with this, *HuR* knockdown in human embryonic kidney 293 (HEK293) cells induces widespread changes in exon usage, further supporting its involvement in alternative splicing [85]. More specifically, nuclear HuR has been implicated in regulating transcripts involved in metabolism and mitochondrial function. In HEK293 cells, HuR binds to a long non-coding RNA (lncRNA) transcribed from the RNA component of mitochondrial RNA processing endoribonuclease (*RMRP*) gene, facilitating its export to the cytoplasm where it regulates mitochondrial DNA replication and RNA processing [86]. Similarly, in activated B cells, HuR regulates expression of the dihydrolipoamide s-succinyltransferase (*Dlst*) gene, a critical component of the alpha-ketoglutarate dehydrogenase (α-KGDH) complex in the tricarboxylic acid (TCA) cycle, by binding to introns within *Dlst* pre-mRNA, thereby preventing the production of non-functional transcript variants [87].

### 5.2. Cytoplasmic Roles of HuR During Stress and Disease

During cellular stress, HuR plays an increasingly prominent role in regulating mature mRNA stability within the cytoplasm [88] (Table 2). Translocation of HuR from the nucleus to the cytoplasm is observed in various pathological and stress-related contexts, including cancer progression [59], oxidative injury [60], inflammation [89,90], fibrosis [40], and cell death [33]. Although a basal level of cytoplasmic HuR is present under homeostatic conditions [91], both its upregulation and increased cytoplasmic localization have been implicated in the pathogenesis of inflammatory diseases such as rheumatoid arthritis [92], atherosclerosis [93], and inflammatory bowel disease [94], as well as in cancer progression [59]. Once in the cytoplasm, HuR stabilizes and enhances the translation of thousands of ARE-containing transcripts, many of which encode cell survival proteins and proinflammatory cytokines [84,95]. Stabilized mRNAs include those encoding key regulators of proliferation—such as *c-FOS*, *CDKN1A*, cyclin B1/D1/E1 (*CCNB1*/*D1*/*E1*), and mouse double minute 2 homolog (*MDM2*)—as well as inflammatory mediators, including inducible nitric oxide synthase (*iNOS* or *NOS2*), granulocyte-macrophage colony-stimulating factor (*GM-CSF* or *CSF2*), tumor necrosis factor (*TNF*), and prostaglandin-endoperoxide synthase 2 (*PTGS2* or *COX2*) [88]. In some cases, HuR enhances the translation of target mRNAs without altering their stability, as seen with X-linked inhibitor of apoptosis (*XIAP*) [96], while in rare instances such as with internal ribosome entry site (IRES)-containing transcripts like cyclin dependent kinase inhibitor 1b mRNA (*CDKN1B* or p27), HuR binding suppresses translation [97].

Beyond its pro-inflammatory and pro-tumorigenic targets, HuR also regulates mRNAs encoding other RNA-binding proteins, mRNA processing factors, transcription factors, and cytokines, reflecting its broad influence on gene expression across disease states [85]. For instance, HuR stabilizes forkhead box Q1 (*FOXQ1*) mRNA in MDA-MB-231 triple-negative breast cancer cells, promoting tumorigenesis and metastases [98], and stabilizes *TGFB* mRNA in activated HSCs, contributing to fibrosis in chronic liver disease [41]. Importantly, cytoplasmic HuR is not always pathogenic. It also supports adaptive cellular responses by stabilizing transcripts that encode metabolic enzymes [42], structural proteins [99], and signaling mediators [100]. In fact, reduced HuR expression is linked to the progression of certain disorders, including obesity and MASLD [42,100–102], underscoring its potential protective role in maintaining cellular homeostasis. Despite these advances, the full extent of HuR’s role in maintaining cellular homeostasis and its contribution to disease pathogenesis remains incompletely understood.

## 6. Role of HuR in Liver Homeostasis and MASLD

### 6.1. MASLD Pathogenesis and Progression

The pathogenesis and progression of MASLD are complex and heterogeneous, with several models proposed to explain its underlying mechanisms. The term non-alcoholic steatohepatitis (NASH) was first introduced in 1980 to describe a form of steatotic liver disease (SLD) that mimics alcoholic hepatitis but occurs in the absence of significant alcohol intake [106]. In 1998, Day and James proposed the ‘two-hit hypothesis,’ which highlighted overlapping mechanisms contributing to SLD regardless of etiology [107]. This model suggested that hepatic steatosis represents the first ‘hit,’ while a second ‘hit’—such as oxidative stress, a common driver across SLD subtypes—is required for progression to steatohepatitis [107]. Although initially applied to both MASLD and alcohol-associated liver disease (ALD), the two-hit hypothesis was ultimately deemed insufficient to capture the multifactorial and dynamic nature of MASLD pathogenesis [108]. Consequently, it was replaced in 2016 by the ‘multiple-hits hypothesis,’ which posits that numerous factors act in concert to drive MASLD progression [108].

The list of contributing mechanisms to MASLD pathogenesis and progression continues to grow, reflecting the increasing recognition of MASLD’s complexity [109]. It is now well established that MASLD pathogenesis begins with hepatic steatosis, driven by an imbalance between lipid acquisition and elimination [110]. In individuals with MASLD, approximately 60% of hepatic lipids originate from adipose tissue lipolysis, 25% from hepatic *de novo* lipogenesis (DNL), and 15% from dietary intake [111]. Both systemic and hepatic insulin resistance play central roles in promoting adipose lipolysis and hepatic DNL [112], while excess dietary intake of fats and fructose further exacerbates hepatic lipid accumulation [113]. Additionally, impaired lipid elimination via very-low-density lipoprotein (VLDL) secretion and fatty acid oxidation (FAO) may contribute more significantly as MASLD progresses [113]. In ~10–30% of patients, MASLD advances to MASH due to the convergence of multiple pathological mechanisms [108]. Lipotoxicity—marked by the accumulation of reactive lipid species in hepatocytes—triggers apoptotic signaling [114,115], damages organelle membranes [116], and induces oxidative stress [117], culminating in hepatocellular injury [113]. This injury activates liver-resident macrophgages and recruits circulating immune cells, amplifying inflammation and tissue damage [113]. Persistent injury and inflammation activate HSCs, leading to extracellular matrix deposition, progressive fibrosis, and ultimately cirrhosis in severe cases [118]. Liver injury and fibrosis are strong predictors of liver-related morbidity and mortality, as well as increased risk of extrahepatic malignancies and CVD in MASLD patients [1]. HCC can arise in both cirrhotic and non-cirrhotic MASH, with contributing mechanisms including chronic inflammation, insulin resistance, lipotoxicity, and cycles of hepatocyte injury and regeneration.

**Figure 2.**
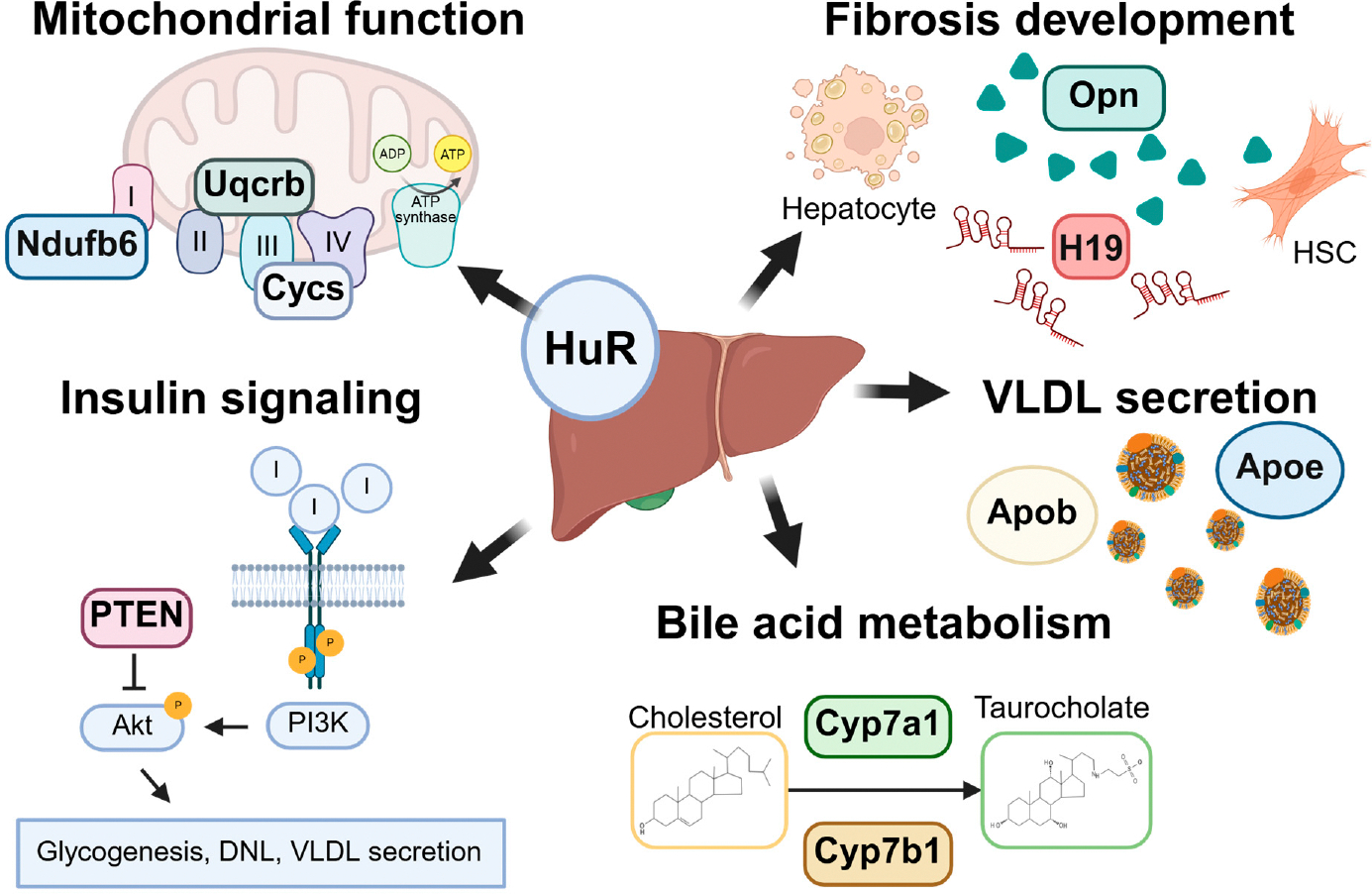
Graphical summary of HuR’s roles in liver homeostasis and MASLD pathogenesis. HuR plays a multifaceted role in maintaining hepatic homeostasis and regulating key processes implicated in MASLD development. These include electron transport chain activity and mitochondrial function, hepatic stellate cell (HSC) activation and fibrogenesis, insulin signaling via the phosphoinositide 3-kinase (PI3K)/AKT serine/threonine kinase 1 (AKT1) pathway, lipoprotein assembly and VLDL secretion, and bile acid synthesis and metabolism. HuR directly or indirectly regulates the expression of a wide array of target transcripts involved in these pathways, including NADH ubiquinone oxidoreductase subunit B6 (*Ndufb6*), ubiquinol–cytochrome c reductase binding protein (*Uqcrb*), cytochrome C (*Cytc*), osteopontin (*Opn*), H19, phosphatase and tensin homolog (*Pten*), apolipoprotein b (*Apob*), apolipoprotein e (*Apoe*), cytochrome P450 family 7 subfamily A member 1 (*Cyp7a1*), and cytochrome P450 family 7 subfamily B member 1 (*Cyp7b1*). Collectively, these regulatory activities position HuR as a key post-transcriptional mediator in both physiological liver function and MASLD progression. Created in BioRender V2. https://BioRender.com/qshr340. (accessed on 1 July 2025).

Although significant progress has been made in understanding the molecular mechanisms underlying MASLD, the factors that drive the disease progression from MASL to MASH in a subset of patients remain incompletely understood. This knowledge gap continues to hinder effective therapeutic development, despite numerous MASLD-related clinical trials currently ongoing [1]. In this context, exploring the role of HuR, a dynamic and multifaceted RNA-binding protein, in a heterogeneous and multisystem disease like MASLD may offer valuable insights. Importantly, the loss of hepatic HuR expression observed in both MASLD patients and preclinical mouse models suggests a potential role for HuR in maintaining liver homeostasis and protecting against MASLD development [101]. Consistently, liver-specific *HuR* knockout mice show increased susceptibility to diet-induced MASLD, highlighting HuR and its downstream pathways as promising therapeutic targets [100–102,119]. The remainder of this review will explore HuR’s regulatory functions in key pathways commonly disrupted in MASLD and its associated metabolic co-morbidities (Figure 2).

### 6.2. Role of HuR in Hepatic Steatosis

#### 6.2.1. HuR Regulates Insulin Signaling and Hepatic Steatosis

MASLD is strongly associated with insulin resistance and hyperinsulinemia [112]. More than 60% of individuals with T2DM also develop MASLD, placing them at significantly elevated risk for progression to pro-fibrotic MASH [120]. Insulin resistance in adipose tissue, liver, and skeletal muscle contributes to the metabolic disturbances that drive hepatic steatosis in MASLD [121]. In adipose tissue, insulin resistance impairs the anti-lipolytic effects of insulin, resulting in sustained lipolysis and increased circulating free fatty acids (FFAs), which promote ectopic lipid deposition in the liver [122]. In parallel, hepatic insulin resistance enhances DNL [121], while insulin resistance in skeletal muscle reduces glucose uptake and glycogen synthesis, leading to systemic hyperglycemia and further stimulating hepatic DNL [123].

Paradoxically, liver-specific *HuR*-deficient mice display enhanced insulin sensitivity and lower blood glucose levels compared to wild-type controls following 24 weeks of high-fat diet (HFD) feeding, despite exhibiting more pronounced hepatic steatosis [100]. To elucidate the underlying mechanisms, hepatic expression and activation of insulin signaling mediators, including components of the phosphoinositide 3-kinase (PI3K)/AKT serine/threonine kinase 1 (AKT1) pathway, were assessed in *HuR*-deficient mice [100]. Under normal conditions, hepatic insulin signaling via the PI3K/Akt axis promotes glucose utilization through glycogenesis, DNL, and triglyceride export, while concurrently inhibiting hepatic glucose production [121]. In *HuR*-deficient livers, Akt activation was elevated, and the expression of genes involved in glycolysis and DNL was increased, while genes related to hepatic glucose production were suppressed—indicating heightened sensitivity to insulin signaling [100]. Phosphatases such as phosphatase and tensin homolog (PTEN) act as negative regulators of the PI3K/Akt pathway and thereby reduce hepatic insulin sensitivity [124]. Like *HuR*-deficient mice, liver-specific *Pten* knockout mice show increased insulin sensitivity but develop more severe hepatic steatosis [125]. PTEN expression was decreased in *HuR*-deficient livers, and *Pten* mRNA was shown to associate with HuR in ribonucleoprotein immunoprecipitation (RNP-IP) assays from hepatocytes [100]. These findings suggest that HuR stabilizes *Pten* transcripts and that its loss leads to reduced *Pten* expression, enhanced PI3K/Akt signaling, and increased hepatic insulin sensitivity, thereby promoting DNL. However, overexpression of PTEN in *HuR*-deficient livers did not reduce serum alanine aminotransferase (ALT) levels following HFD feeding, indicating that loss of *HuR*-mediated PTEN stabilization does not fully explain the susceptibility to liver injury in this model [100]. Supporting this, two additional studies using liver-specific *HuR* knockout mice reported significant upregulation of DNL-related genes, such as acetyl-CoA carboxylase 1 (*Acc1*) and fatty acid synthase (*Fasn*), compared to wild-type controls, under both normal chow [101] and Western diet (42 kcal% fat (62% saturated), 0.1% cholesterol) plus sucrose water (WDSW) feeding conditions [102]. These findings collectively suggest that hepatic HuR plays a critical role in modulating insulin signaling and lipid metabolism, where its loss enhances insulin sensitivity but simultaneously promotes hepatic lipogenesis and steatosis.

#### 6.2.2. HuR Regulation of Very Low-Density Lipoprotein Secretion

The assembly and secretion of VLDL particles represent the primary pathway for hepatic lipid export and are essential for maintaining liver lipid homeostasis [126]. Impaired VLDL secretion leads to lipid accumulation, lipotoxicity, and exacerbation of MASLD [127–129]. In hepatocytes, VLDL particles are synthesized through microsomal triglyceride transfer protein (MTTP)–mediated lipidation of apolipoprotein B-100 (APOB-100), followed by the incorporation of storage lipids such as triglycerides and cholesterol esters [126]. Additional apolipoproteins, including apolipoprotein E (APOE), contribute to the stabilization and distribution of VLDL particles [130]. Enhanced VLDL secretion is generally protective against hepatic steatosis, as evidenced by the increased prevalence of MASLD in individuals with loss-of-function mutations in *MTTP* or *APOB* [131]. However, chronic overproduction of VLDL in MASLD patients is also linked to dyslipidemia and heightened cardiovascular risk [11,127].

In liver-specific *HuR*-deficient mice, increased hepatic steatosis was accompanied by reduced expression of both APOB-100 and APOE, as well as decreased serum lipid levels [119]. Supporting a direct regulatory role for HuR, the authors demonstrated HuR binding to *Apob* pre-mRNA, which enhanced APOB expression in a human hepatoma cell line [119]. Similarly, another study using the same Alb-Cre-driven *HuR* knockout model found elevated hepatic steatosis under normal chow conditions, though this effect was not observed after 6 weeks of choline-deficient high-fat diet (HFD-CD) feeding [101]. Because choline deficiency impairs hepatic VLDL secretion [132], this dietary context may have masked the effect of *HuR* loss on lipid export. Further supporting a role for HuR in VLDL regulation, shotgun lipidomic analysis revealed increased levels of major VLDL components such as various triglyceride and cholesterol ester species in *HuR*-deficient livers, with lipid profiles resembling those observed in human MASLD [101]. In addition, endoplasmic reticulum (ER) stress and dysregulated FXR signaling—both known to impair VLDL secretion and promote steatosis in MASLD [126]—were also observed in *HuR*-deficient livers [101]. Taken together, these studies support a critical role of HuR in VLDL secretion.

### 6.3. Role of HuR in Cholesterol Metabolism

While overall hepatic fat accumulation defines MASLD, dysregulation of cholesterol metabolism is increasingly recognized as a critical driver of disease progression [133]. Cholesterol homeostasis in the liver is governed by a balance between assimilation (uptake, synthesis, esterification, storage) and elimination (lipoprotein secretion, bile acid synthesis, and excretion) [133]. In human MASLD, hepatic cholesterol accumulation is frequently linked to increased endogenous cholesterol synthesis [133]. This process is regulated by sterol regulatory element-binding protein 2 (*SREBP-2*, *SREBF2*), an ER-resident transcription factor activated under low sterol conditions [134]. Hepatic cholesterol biosynthesis involves a multi-step pathway, beginning with the conversion of acetyl-CoA to mevalonate by hydroxymethylglutaryl-CoA reductase (*HMGCR*/*HMGCR*), the rate-limiting step of the pathway and a major control point [135]. Cholesterol metabolites, glucagon, and statins inhibit HMGCR, whereas insulin and SREBP-2 activate HMGCR [135,136]. While hepatic cholesterol accumulation normally suppresses SREBP-2 activation, inflammation and insulin resistance can override this feedback, resulting in sustained cholesterol synthesis during MASLD [137,138].

Paradoxically, although *HuR*-deficient mouse livers show increased hepatic cholesterol content under MASLD-inducing diets [101,102], transcriptomic analysis revealed that cholesterol synthesis pathways were significantly downregulated in *HuR*-deficient livers under normal dietary conditions [101]. In the same study, RNP-IP sequencing (GEO access ID: GSE143703) in healthy murine liver tissue revealed direct binding of HuR to the *Hmgcr* mRNA, indicative of HuR-mediated post-transcriptional regulation of *Hmgcr*. These findings suggest that HuR loss alters the regulatory landscape of cholesterol metabolism even before overt MASLD develops, though further investigation is needed to clarify how HuR influences *Hmgcr* expression and cholesterol synthesis under different dietary conditions.

Following synthesis, cholesterol can be stored or utilized in various cellular processes, including bile acid (BA) synthesis and steroidogenesis. Excess free cholesterol is esterified by acyl-CoA: cholesterol acyltransferase (ACAT) for storage in lipid droplets [139]. Alternatively, unesterified cholesterol is distributed to cellular membranes to maintain membrane fluidity and serve as a precursor for cholesterol derivatives [139]. In hepatocytes, free cholesterol predominantly accumulates in the plasma membrane, while its accumulation in organelle membranes, particularly the mitochondrial and ER membranes, is tightly restricted to preserve membrane integrity [135]. During MASLD, excess accumulation of free cholesterol in the mitochondrial and ER membranes leads to mitochondrial dysfunction and ER stress, promoting inflammation, cell death, and MASLD progression [116]. Although membrane-specific free cholesterol distribution has not yet been assessed in *HuR*-deficient mouse livers, elevated total hepatic cholesterol levels in these mice are accompanied by increased mitochondrial dysfunction [119] and ER stress [101], raising the possibility that disrupted cholesterol compartmentalization, including excess free cholesterol, contributes to liver injury in this model. This warrants further investigation into the role of HuR in regulating intracellular cholesterol distribution and its implications for MASLD progression.

### 6.4. Role of HuR in Bile Acid Metabolism

MASLD development is associated with a progressive increase in serum bile acid levels and alterations in bile acid pool composition [140,141]. The hepatic pathways responsible for the conversion of cholesterol into bile acids, including cholic acid (CA) and chenodeoxycholic acid (CDCA), have been extensively characterized [142]. However, the underlying mechanisms driving bile acid induction in MASLD remain controversial and incompletely understood [143]. For instance, in two MASLD cohorts, enlarged bile acid pool size was associated with both induction of bile acid synthesis genes, including cytochrome P450 family 7 subfamily A member 1 (*CYP7A1*), cytochrome P450 family 8 subfamily B member 1 (*CYP8B1*), and cytochrome P450 family 27 subfamily A member 1 (*CYP27A1*) [144] and increased serum levels of bile acid synthesis marker 7-alpha-hydroxy-4-cholesten-3-one (C4) [145]. In contrast, increasing serum bile acid levels in another MASLD cohort were not associated with changes in bile acid synthesis gene expression or C4 levels [140]. Similar discrepancies have been observed in rodent models of MASLD. For instance, in rats, MASLD induction led to increased expression of *Cyp7a1* and *Cyp8b1* [144], whereas a MASLD mouse model showed elevated hepatic bile acid levels despite decreased expression of classical bile acid synthesis genes, including *Cyp27a1* and *Cyp8b1* [146]. As bile acid metabolism is regulated by a complex interplay of factors, including interspecies differences in the expression of bile acid synthesis genes [142], gut microbiome composition [147], sex-specific variations [148], and dietary influences [148], these factors contribute to variations in bile acid pool composition and may account for the discrepancies observed across studies.

HuR appears to play a regulatory role in hepatic BA synthesis [101,102]. In hepatocyte- and cholangiocyte-specific *HuR*-deficient mouse livers, key genes involved in both classical and alternative BA synthesis pathways, such as *Cyp7a1* and *Cyp7b1*, are downregulated. RNP-IP from healthy murine liver tissue confirms that HuR directly binds to both *Cyp7a1* and *Cyp7b1* transcripts, suggesting post-transcriptional regulation by HuR [101]. Cyp7b1 repression is commonly observed during MASLD and is associated with the induction of the alternative BA synthesis pathway, leading to oxysterol accumulation, which contributes to hepatic inflammation and fibrosis [149–151]. Whether Cyp7b1 suppression in *HuR*-deficient livers promotes oxysterol accumulation during MASLD remains to be determined. In addition to gene repression, hepatocyte- and cholangiocyte-specific *HuR*-deficient livers show reduced levels of conjugated primary bile acids, including taurochenodeoxycholic acid (TCDCA) and tauroursodeoxycholic acid (TUDCA), relative to their wild-type counterparts [101]. In contrast, hepatocyte-specific *HuR*-deficient mice exhibit increased levels of conjugated primary and secondary bile acids in both liver and serum following MASLD-inducing diet feeding [102]. Wang, Y. et al. hypothesized that accumulation of bile acids in *HuR* deficient mouse livers may promote MASH fibrosis by activating sphingosine-1-phosphate receptor 2 (S1PR2) and upregulating expression of the lncRNA, H19 [102]. The discrepancies between these two studies may be attributed to differences in the scope of *HuR* deletion (hepatocyte-specific vs. hepatocyte-and-cholangiocyte-specific) as well as variations in diet feeding conditions.

Enterohepatic bile acid circulation, which recycles bile acids between the liver and the intestine, is essential not only for the disposition of lipid-soluble metabolites, but also for bile acid-mediated regulation of metabolic homeostasis [152]. Around 95% of the bile acids that enter the small intestine are reabsorbed by ileal enterocytes. Prior to recycling, some bile acids are metabolized by gut bacteria in the distal ileum and large intestine into secondary BAs, including deoxycholic acid (DCA), hyocholic acid (HCA), lithocholic acid (LCA), ursodeoxycholic acid (UDCA), and omega-muricholic acid (ω-MCA) [152]. Indicative of impaired enterohepatic bile acid circulation in *HuR*-deficient mice, conjugated bile acids, including tauro-CA (TCA), are elevated in the liver and serum of hepatocyte-specific *HuR*-deficient mice compared to wild-type controls, while primary and secondary bile acids are reduced in the cecum following MASLD-inducing diet feeding [102]. In summary, these findings suggest a multifaceted role for HuR in regulating bile acid metabolism, including regulation of BA synthesis and enterohepatic circulation, potentially contributing to MASLD progression.

### 6.5. HuR Regulates Mitochondrial Function and Oxidative Stress

Mitochondrial dysfunction is a key contributor to MASLD progression [117]. As central regulators of lipid metabolism and cellular energy homeostasis, mitochondria can both protect the liver from hepatic steatosis and promote MASLD progression during periods of prolonged metabolic stress [153,154]. For instance, mitochondrial FAO is initially upregulated in response to increased intrahepatic lipid accumulation during early MASLD development [153]. However, as the disease progresses to MASH, mitochondrial oxidative metabolism becomes impaired, resulting in reduced ATP production and increased reactive oxygen species (ROS) generation [153–155]. Accumulation of ROS in dysfunctional mitochondria contributes to oxidative injury in MASLD [156]. To mitigate oxidative stress, the nuclear factor erythroid 2-related factor 2 (NRF2, *NFE2L2*) pathway is activated, inducing the expression of key cytoprotective genes such as superoxide dismutase 2 (*SOD2*) and heme oxygenase-1 (HO-1, *HMOX1*) [157].

HuR appears to play a hepatocyte-specific role in regulating mitochondrial function and the oxidative stress response. For instance, following 4 weeks of HFD feeding, increased hepatic steatosis in liver-specific *HuR*-deficient mice was associated with significantly reduced ATP levels and the downregulation of electron transport chain proteins, including cytochrome c (Cytc), NADH: ubiquinone oxidoreductase subunit B6 (Ndufb6), and ubiquinol–cytochrome c reductase binding protein (Uqcrb) [119]. Direct binding of HuR to the mRNAs of *Cytc*, *Ndufb6*, and *Uqcrb* was also demonstrated in mouse hepatoma Hepa1–6 cells, supporting a post-transcriptional regulatory role for HuR in mitochondrial function [119]. However, the overexpression of Cytc alone did not restore mitochondrial ATP production in *HuR*-deficient livers, suggesting that HuR may influence mitochondrial function through multiple pathways [119]. Regulation of oxidant scavenging by HuR may be one additional mechanism by which HuR responds to mitochondrial dysfunction during MASLD progression. For instance, HuR-mediated upregulation of *HMOX1* is associated with improved survival in liver transplant recipients [60]. Furthermore, in rats fed a methionine- and choline-deficient (MCD) diet for six weeks, a positive correlation between hepatic HuR expression and both *Hmox1* and *Sod2* expression was observed, further supporting HuR’s role in regulating oxidative stress responses in vivo [158].

In summary, HuR helps preserve mitochondrial function and protects against oxidative injury by regulating components of the electron transport chain and key antioxidant defense pathways, highlighting its potential role in preventing MASLD progression. Mitochondrial quality control and dynamics—regulated by dynamin-related protein 1 (DRP1), mitofusins 1 and 2 (MFN1/2), optic atrophy 1 (OPA1), and the PTEN-induced kinase 1/parkin RBR E3 ubiquitin protein ligase (PINK1/Parkin) pathway—are critically important in MASLD pathogenesis [159–161]. Disruptions in these pathways exacerbate mitochondrial dysfunction, inflammation, and metabolic stress, thereby accelerating MASLD progression [162–164]. Although HuR likely contributes to mitochondrial maintenance through post-transcriptional regulation in hepatocytes, direct evidence linking HuR to the regulation of mitochondrial quality control and fission/fusion dynamics in MASLD is currently limited. This remains a promising area for future investigation.

### 6.6. Role of HuR in Autophagy

Autophagic degradation of lipids (lipophagy) and damaged mitochondria (mitophagy) plays a protective role in preventing MASLD progression to MASH [165]. Although autophagy is constitutively active in all cell types, it is strongly induced under conditions of cellular stress and energy depletion [166]. During autophagy, autophagy-related proteins, including Atg8/microtubule-associated protein 1A/1B light chain (LC3) and selective autophagy receptors (SARs) such as sequestosome 1 (SQSTM1/p62), target cellular components for sequestration into double-membraned vesicles called autophagosomes [165]. Fusion of autophagosomes with lysosomes enables degradation of the sequestered materials [166]. In autophagy-deficient livers, clearance of lipid droplets and dysfunctional mitochondria is impaired, contributing to increased hepatic steatosis and mitochondrial dysfunction during MASLD progression [166,167].

While HuR’s specific role in autophagy during MASLD remains to be defined, growing evidence suggests that HuR functions as a positive regulator of autophagy in various cell types and disease contexts, particularly under oxidative and metabolic stress [168]. In liver cancer cell lines such as L-02 and Hep3B, *HuR* deficiency reduces autophagosome formation and autophagic flux. RNP-IP revealed direct HuR binding to the 3′ UTRs of autophagy proteins: *Atg5*, *Atg12*, and *Atg16* mRNAs in Hep3B cells [169]. Similarly, in *HuR*-deficient mouse livers, reduced expression of Atg3, Atg5, and Atg7 was observed alongside more severe liver injury following acetaminophen (APAP) overdose, and the direct binding of HuR to the 3′ UTRs of these genes was also confirmed in Hepa1–6 cells [170]. Given HuR’s established role in regulating autophagy across multiple tissues and models of liver injury, further investigation into its function in autophagy during MASLD development is highly warranted.

### 6.7. HuR Regulates Cell Death and Survival

Apoptosis, necroptosis, pyroptosis, and ferroptosis are distinct forms of regulated cell death that play critical roles in tissue homeostasis, inflammation, and disease progression. Apoptosis is a programmed, non-inflammatory form of cell death characterized by cell shrinkage, chromatin condensation, and caspase activation, typically involved in tissue remodeling and the removal of damaged cells [171]. In contrast, necroptosis is a form of regulated necrosis triggered by death receptors and mediated by receptor-interacting proteins 1 and 3 (RIPK1 and RIPK3) and mixed lineage kinase domain-like (MLKL), leading to cell swelling, membrane rupture, and the release of pro-inflammatory contents [172]. Pyroptosis is a highly inflammatory form of lytic cell death initiated by inflammasome activation and caspase-1/11, resulting in cleavage of gasdermin D and release of cytokines such as IL-1β and IL-18 [173]. Ferroptosis is a non-apoptotic form of cell death driven by iron-dependent lipid peroxidation, characterized by the accumulation of ROS and depletion of glutathione peroxidase 4 (GPX4) activity [174]. These pathways are increasingly recognized for their contributions to liver injury, inflammation, and fibrosis in chronic liver diseases, including MASLD [175].

Apoptosis is considered the predominant mode of regulated cell death in MASLD livers, and caspase substrates, such as cytokeratin-18, have been proposed as potential serum biomarkers for human MASH [113,176]. HuR, widely recognized as a survival-promoting factor in various cancers, regulates the stability of transcripts encoding both pro-apoptotic proteins (e.g., p53, p27, Caspase-8, Caspase-9, FAS, c-Myc) and anti-apoptotic proteins (e.g., B-cell lymphoma 2 [BCL2], Sirtuin 1 [SIRT1], prothymosin α [PTMA]) [177–179]. However, under conditions of lethal cellular stress, HuR itself is cleaved and adopts a pro-apoptotic role by directly binding to and stabilizing *CASP9* mRNA [33,34].

Apoptosis induction in MASLD is strongly associated with the accumulation of non-storage lipids, including FFAs and free cholesterol, in the liver [114,115]. Although HuR’s specific role in hepatocyte apoptosis during MASLD remains to be fully elucidated, elevated hepatic FFA and cholesterol levels have been observed in *HuR*-deficient mouse livers, correlating with increased liver injury after 24 weeks of high-fat diet feeding [100]. These non-storage lipids contribute to hepatocyte apoptosis by activating both intrinsic (organelle-mediated) and extrinsic (death receptor-mediated) apoptotic pathways [116,180,181]. Intrinsic apoptosis is initiated when FFAs and free cholesterol accumulate in organelle membranes, particularly the ER, mitochondria, and lysosomes, triggering ER stress, mitochondrial dysfunction, and release of lysosomal proteases [180]. RNP-IP analysis from healthy murine livers revealed direct binding of HuR to transcripts involved in the ER stress response, suggesting a potential role for HuR in regulating organelle stress pathways during MASLD [101]. In parallel, non-storage lipids also activate extrinsic apoptosis signaling. FFAs sensitize hepatocytes to apoptosis via c-JUN N-terminal kinase (JNK) activation, upregulation of death receptor 5 (DR5) and Fas receptor, and activation of Bax [182,183]. Similarly, excessive free cholesterol accumulation within mitochondrial membranes increases mitochondrial sensitivity to oxidative stress and enhances hepatocyte susceptibility to TNFα- and Fas ligand-mediated apoptosis [116]. Both DR5 and Fas are highly expressed in human MASH livers, which also exhibit greater degrees of mitochondrial dysfunction [114,182,184]. In liver cancer models, HuR has been shown to inhibit apoptosis by directly binding to the 3′ UTR of Fas mRNA in HepG2 cells and repressing its translation [62]. Whether HuR plays a similar anti-apoptotic and survival-promoting role in hepatocytes during MASLD progression remains an important area for future investigation.

In addition to its known role in regulating apoptosis, HuR may also influence other forms of regulated cell death— necroptosis, pyroptosis, and ferroptosis—that contribute to liver injury and inflammation in MASLD. Although direct evidence linking HuR to these pathways in MASLD models is currently limited, emerging studies suggest HuR plays a role in ferroptosis by stabilizing mRNAs of key antioxidant and iron metabolism regulators, such as solute carrier family 7 member 11 (*Slc7a11*), thereby suppressing lipid peroxidation and ferroptotic cell death in cancer models [185,186]. Given the heightened oxidative stress and inflammatory environment characteristic of MASLD, HuR dysregulation may exacerbate disease progression by disrupting the balance among these cell death pathways. Further investigation is needed to elucidate HuR’s mechanistic involvement in apoptosis, necroptosis, pyroptosis, and ferroptosis during MASLD development and progression, and to evaluate its potential as a therapeutic target in MASLD.

### 6.8. Role of HuR in Liver Inflammation

In MASLD, damage-associated molecular patterns (DAMPs) released from stressed or dying hepatocytes and pathogen-associated molecular patterns (PAMPs) from gut-derived microbial products activate Toll-like receptors (TLRs) on various hepatic cell types to initiate immune responses [187]. For instance, TLR4 activation by FFAs and unesterified cholesterol triggers pro-inflammatory signaling cascades, particularly in macrophages [188]. This leads to increased expression of pro-inflammatory cytokines and chemokines such as TNF, interleukin 6 (IL6), interleukin 1β (IL1B), and C-C motif chemokine ligand 2 (CCL2), the molecules tightly linked to MASLD progression and fibrosis [187]. TNF acts on both parenchymal and non-parenchymal cells to promote immune cell recruitment, activation, and fibrogenic remodeling in the liver [187,189]. HuR has been shown to bind directly to the *Tnf* mRNA 3′ UTR in RAW 264.7 macrophages, and its expression is essential for LPS-induced TNF production in bone marrow-derived macrophages [105,190,191]. Additional pro-inflammatory gene targets stabilized by HuR in humans include *CCL2*, *NOS2,* vascular endothelial growth factor (*VEGFA*), and *COX2* [192], while increased HuR expression is associated with *Il6* induction in a murine model of periodontal disease [193].

Although HuR is generally regarded as a positive regulator of inflammation, studies in myeloid-specific *HuR* knockout models suggest a more nuanced role. In these models, *HuR* depletion exacerbates inflammation and increases mortality in response to LPS-induced endotoxemia and chemically induced colitis [194–196]. These seemingly conflicting observations highlight the complex and multifaceted nature of post-transcriptional gene regulation. The interplay between RNA-binding proteins, non-coding RNAs, and context-dependent signals ultimately shapes gene expression outcomes at the post-transcriptional level [197–199]. As an example, tristetraprolin (TTP) is an mRNA-binding protein that, like HuR, binds to AREs in the 3′ UTR of many labile mRNAs [200]. However, unlike HuR, which stabilizes target transcripts, TTP promotes their rapid degradation [200]. In the context of inflammation, both HuR and TTP target pro-inflammatory transcripts such as *TNF* in macrophages [191]. The importance of this regulatory balance is underscored by findings that *Ttp*-deficient mice develop spontaneous systemic inflammation, primarily due to persistent *Tnf* overexpression [201]. While the role of myeloid HuR in MASLD has not yet been studied, hepatocyte-specific *HuR*-deficient mice fed a WD diet for four weeks exhibited increased liver inflammation, characterized by F4/80-positive macrophage infiltration and upregulation of *Ccl2*, *Tnf*, *Il6*, and *Il1b* [102].

Beyond macrophages, the roles of other immune cells, including natural killer T (NKT) cells and adaptive immune populations, are gaining recognition in MASLD pathogenesis [202]. NKT cells exert both pro- and anti-inflammatory effects but are enriched in the liver during MASLD progression to MASH and fibrosis [202,203]. Adaptive immune subsets, including Th1, Th17, and B cells, also increase in abundance and activity with worsening disease severity [202]. HuR has been shown to influence the adaptive immune response; *HuR*-deficient T and B cells exhibit impaired activation and proliferation upon inflammatory stimulation [87,204,205]. Moreover, postnatal *HuR* depletion results in apoptosis of immune progenitor cells in the bone marrow and thymus, highlighting HuR’s essential role in immune cell survival [32]. In the context of MASLD, inflammation and tumorigenesis are exacerbated in hepatocyte- and cholangiocyte-specific *HuR*-deficient mice after 14 months of HFD-CD feeding [101]. These livers show increased infiltration of NKT cells and CD8^+^ T cells, immune subsets commonly associated with severe inflammation and fibrosis in MASLD [101,202,203].

In summary, HuR exerts a context-dependent and multifaceted influence on inflammation, regulating both innate and adaptive immune responses. Its specific roles within individual immune cell populations in the context of MASLD remain unexplored. Further investigation is warranted to elucidate how immune cell-specific HuR activity contributes to immune-mediated mechanisms underlying MASLD progression.

### 6.9. Role of HuR in Liver Fibrosis

Fibrosis is the major predictor of liver-related morbidity and mortality in MASLD patients, significantly increasing the risk of liver failure and HCC [206,207]. Hepatic fibrosis develops when chronic inflammation, sustained cell death, and aberrant wound healing activate normally quiescent fibroblasts, primarily HSCs, into highly proliferative, contractile, and collagen-secreting myofibroblasts [118,208]. HSC activation is driven by various profibrotic signals, including TGF-β and platelet-derived growth factor (PDGF) [208,209]. In MASLD, free cholesterol accumulation within HSCs enhances their activation, while other pro-fibrotic cues, such as Notch signaling activation in hepatocytes and lncRNA H19 induction in cholangiocytes, further amplify fibrogenic responses [102,210–212].

HuR plays a complex and cell-type-specific role in liver fibrosis. In hepatocytes, HuR appears protective, as its deficiency exacerbates fibrosis progression in MASLD mouse models [101,102]. Hepatocyte- and cholangiocyte-specific *HuR* deficiency leads to hepatic cholesterol accumulation and marked dysregulation of cholesterol metabolism, conditions known to promote HSC activation [101]. These *HuR*-deficient mice also showed increased liver fibrosis, accompanied by elevated expression of the Notch signaling mediator osteopontin (OPN) [101] and lncRNA H19 under WDSW diet feeding [102].

In contrast, HuR expression in hepatic myofibroblasts (activated HSCs) promotes both bile duct ligation and CCl_4_-induced fibrosis development [40,41]. In murine HSCs, TGF-β1 and PDGF both induce cytoplasmic localization of HuR, while PDGF specifically increases HuR expression [40,41]. In *HuR*-deficient rat HSCs, PDGF-induced proliferation and migration are significantly impaired [40]. RNP-IP experiments in the activated HSC line CFSC-8B revealed that HuR binds directly to transcripts encoding pro-proliferative (e.g., cyclin D1, cyclin B1) and motility-related proteins (e.g., matrix metallopeptidase 9 [MMP9], α-SMA) following PDGF stimulation [40]. Similarly, HuR directly binds to sphingosine kinase 1 (*SPHK1*, *Sphk1*) mRNA, which is required for TGF-β1-mediated induction of fibrillar collagen 1a1 (*COL1A1*, *Col1a1*) and smooth muscle actin (*ACTA2*, *Acta2*) expression in both human HSCs LX-2 cells and murine primary HSCs [41]. In addition to promoting HSC activation, HuR may also contribute to fibrosis resolution. In a recent study, sorafenib-induced ferroptosis in HSCs was dependent on HuR-mediated stabilization of the autophagy gene beclin-1 (*BECN1*), suggesting a role for HuR in promoting autophagy and cell death in activated HSCs under therapeutic stress [213].

In summary, HuR functions as a pivotal post-transcriptional regulator of liver fibrosis, with cell-type–specific effects that may drive both the progression and resolution of fibrogenic responses in MASLD. Future research should aim to dissect the signaling networks through which HuR modulates HSC plasticity, with the goal of identifying new therapeutic strategies to prevent or reverse hepatic fibrosis.

## 7. Role of HuR in Extrahepatic Metabolic Comorbidities

MASLD is the hepatic manifestation of the metabolic syndrome and is thus closely linked to a range of other metabolic disorders, including obesity, T2DM, and CVD [1]. Reflecting the central role of systemic metabolic dysfunction in MASLD pathogenesis, current diagnostic criteria require the presence of at least one additional cardiometabolic risk factor, such as obesity or insulin resistance, in conjunction with hepatic steatosis to establish a MASLD diagnosis [7]. In this section, we provide a brief overview of HuR’s emerging roles in extrahepatic metabolic diseases commonly associated with MASLD, highlighting its potential contribution to the broader metabolic syndrome context.

### 7.1. Obesity

#### 7.1.1. Overview of Obesity and Its Association with MASLD

Obesity is a well-established independent risk factor for MASLD [214]. While chronic low-grade inflammation, insulin resistance, and dyslipidemia are recognized contributors to MASLD development in obese individuals, the molecular drivers of these pathophysiological changes remain incompletely defined [215]. For instance, a clearly established link exists between dysregulation of adipokine signaling and MASLD development [214]; however, what regulates the expression and secretion of adipokines, including leptin and adiponectin, in the context of obesity is incompletely understood [216]. Given these knowledge gaps, investigating HuR’s role in adipose tissue function and obesity development may offer novel insights into the pathogenesis of MASLD and reveal new therapeutic targets.

#### 7.1.2. Role of HuR in Adipocyte Differentiation and Obesity

HuR is a key post-transcriptional regulator of adipocyte function, and its expression is dynamically regulated during adipocyte differentiation and obesity development. In vitro differentiation of 3T3-L1 pre-adipocytes is associated with increased HuR expression and cytoplasmic localization [31]. In contrast, in vivo studies show that mature adipocytes express lower levels of HuR compared to pre-adipocytes residing in the stromal vascular fraction [42]. Importantly, obesity is associated with further suppression of HuR expression in adipose tissue in both humans and mice [42]. In this section, we highlight HuR’s role in adipocyte differentiation and dysfunction, with an emphasis on mechanisms relevant to MASLD [31,42,43,217–219].

Adipocyte differentiation from pre-adipocytes into mature and lipid-storing adipocytes is essential for maintaining lipid homeostasis [215]. HuR supports this process by stabilizing mRNAs encoding key adipogenic regulators, including CCAAT/enhancer-binding protein beta (*C/EBPβ*) and solute carrier family 2 member 1 (*SLC2A1*, also known as *GLUT1*) [31]. In addition, HuR directly stabilizes transcripts encoding peroxisome proliferator-activated receptor gamma (*PPARG*) and adiponectin (*ADIPOQ*), both of which are critical for adipocyte function and insulin sensitivity [217]. The activity and localization of HuR during adipocyte differentiation are modulated by regulatory signals, including signaling mediated by *PPARG* and the lncRNA *CAAlnc1*. For instance, binding of *CAAlnc1* to HuR protein interferes with HuR-mediated stabilization of adipogenic transcription factor mRNAs in C3H10 cells [219].

In vivo, HuR is essential for maintaining adipose tissue health and systemic metabolic balance. Adipocyte-specific *HuR* deletion in mice leads to exacerbation of obesity, MASLD, and CVD [42,43,218]. Following 16 weeks of high-fat diet feeding, adipocyte-specific *HuR*-deficient mice exhibited significantly greater hepatic steatosis than wild-type controls. This was partly attributed to reduced expression of patatin-like phospholipase domain-containing 2 (*Pnpla2*), which encodes adipose triglyceride lipase (ATGL), a key enzyme in adipocyte lipolysis [42]. Impaired ATGL activity resulted in adipocyte hypertrophy, inflammation, and systemic insulin resistance, all of which promote MASLD progression. Further studies demonstrated that adipocyte-specific *HuR* deficiency selectively impacted abdominal (epididymal) adipose depots, but not subcutaneous (inguinal) depots [43]. This regional HuR loss was associated with increased adipogenesis and pro-inflammatory cytokine expression, a pattern closely linked to obesity-associated MASLD in humans [43]. In the context of CVD, *HuR* deficiency in adipocytes led to cardiac hypertrophy and fibrosis, despite unaltered HuR expression in heart tissue. Transcriptomic analyses revealed significant alterations in both adipose and cardiac gene expression, and the pro-inflammatory environment in *HuR*-deficient adipose tissue likely contributed to a systemic inflammatory state, promoting pathological cardiac remodeling [218].

In summary, adipocyte-specific HuR is a critical regulator of adipocyte differentiation, lipid metabolism, and systemic inflammation. Its loss not only promotes adipose tissue dysfunction and obesity but also contributes to the development of MASLD and CVD, underscoring HuR’s central role in metabolic health [42,43,218].

### 7.2. Cardiovascular Disease and Type II Diabetes Mellitus

#### 7.2.1. Overview of Cardiovascular Disease, Diabetic Cardiomyopathy, and Their Link to MASLD

Similarly to obesity, T2DM and MASLD are closely interconnected metabolic disorders [220]. While approximately 25–30% of the global adult population is estimated to have MASLD, the prevalence rises to nearly 60% among individuals with T2DM, underscoring their frequent coexistence [1]. Key pathophysiological contributors to this relationship include systemic insulin resistance and hyperglycemia, both hallmark features of T2DM [121]. Conversely, growing evidence suggests that MASLD itself may act as a precursor to T2DM, rather than merely a consequence [221]. Mechanistically, hepatic steatosis contributes to systemic insulin resistance, chronic low-grade inflammation, and dyslipidemia, all of which drive T2DM pathogenesis. Moreover, progression of MASLD to MASH and advanced fibrosis further increases the risk for developing T2DM [221].

CVD, including myocardial infarction, ischemic stroke, and heart failure, is the leading cause of death in patients with both MASLD [222] and T2DM [223]. In T2DM, chronic hyperglycemia-induced vascular injury underlies multiple cardiovascular complications, including atherosclerotic cardiovascular disease and diabetic cardiomyopathy [224]. In diabetic cardiomyopathy, insulin resistance and hyperglycemia contribute to persistent inflammation, oxidative stress, cardiomyocyte death, and cardiac remodeling, leading to heart failure independent of atherosclerosis [225]. However, atherosclerosis frequently co-occurs with T2DM and contributes to the 2-fold or greater increase in CVD risk observed in this population [223]. Similarly, MASLD contributes to CVD development through shared mechanisms, including hepatic steatosis–induced insulin resistance, chronic systemic inflammation, and atherogenic dyslipidemia [10]. These overlapping metabolic disturbances suggest that MASLD is not only a liver disease but also a significant contributor to cardiometabolic risk.

#### 7.2.2. Role of HuR in Metabolic Dysfunction-Associated Cardiovascular Disease

HuR expression and cytoplasmic localization in cardiac tissue are associated with CVD development in both human patients and experimental animal models [192,226]. While some discrepancies in the literature exist regarding the direction of HuR’s effects [227], the majority of studies suggest that HuR induction contributes to cardiac dysfunction in conditions such as ischemia/reperfusion (I/R) injury [228–230], pressure overload–induced cardiac remodeling [226,231], and myocardial infarction [232]. In diabetic (db/db) mice, HuR is upregulated not only in cardiomyocytes but also in infiltrating immune cells within the heart [230]. Correspondingly, elevated HuR expression has been observed in myocardial tissue from heart failure patients [226,230], including those with diabetes [233].

HuR contributes to CVD pathogenesis through several mechanisms, including cardiomyocyte death, inflammatory cytokine production, and immune cell infiltration, all of which drive adverse cardiac remodeling and progression to heart failure [225]. Following ischemic injury, HuR induction in the infarct zone is associated with increased expression of pro-inflammatory cytokines such as TNF, IL1B, CCL2, and the pro-apoptotic factor p53. In mice with cardiomyocyte-specific *HuR* depletion, infarct-associated cell death and inflammation were significantly reduced compared to controls [232]. In a coronary artery–ablation model of murine I/R injury, pharmacological inhibition of HuR led to reduced *Il6* and *Tnf* expression in the infarcted region [228]. Interestingly, while cardiomyocyte apoptosis was not different between treatment groups, CD68^+^ immune cell infiltration into the infarct site was reduced in HuR-inhibited mice, correlating with improved cardiac function and attenuated fibrosis [228]. Prolonged inflammation contributes to fibrotic remodeling and heart failure [225]. In diabetic patients with heart failure, an increased number of HuR^+^ and F4/80^+^ macrophages has been observed in failing hearts [230]. In mouse models, macrophage-specific *HuR* deletion led to reduced pro-inflammatory cytokine expression and fibrosis in an angiotensin II–induced model of cardiac injury [230]. Similarly, fibroblast-specific *HuR* deletion resulted in decreased myofibroblast activation (e.g., reduced α-SMA expression), less fibrosis, and preserved cardiac function following pressure overload–induced injury [231]. In a separate study, cardiomyocyte-specific *HuR* deletion protected against cardiac hypertrophy and fibrosis in a pressure overload model, in part by suppressing TGF-β expression in cardiomyocytes [226].

In summary, HuR contributes to the development and progression of CVD under conditions of metabolic stress by promoting inflammation, cell death, immune infiltration, and fibrotic remodeling in the heart. Its cell-specific functions in cardiomyocytes, macrophages, and fibroblasts highlight HuR as a multifaceted regulator of cardiac pathology in metabolic disease contexts such as MASLD and T2DM.

## 8. Conclusions and Future Perspectives

As a ubiquitously expressed and highly dynamic regulator of post-transcriptional gene expression, HuR plays a critical role in numerous cellular processes. Expanding our understanding of HuR function in the context of MASLD holds significant potential for informing the development of novel therapeutic strategies. However, HuR’s essential role in maintaining homeostasis across multiple tissues and cell types presents a major challenge for its therapeutic targeting. This complexity is exemplified by the opposing effects of HuR in different liver cell populations—while hepatocyte-specific HuR protects against diet-induced MASLD progression [101], HuR activation in HSCs promotes fibrogenesis and chronic liver injury [40]. Thus, indiscriminate modulation of HuR across liver cell types could worsen disease outcomes or yield limited benefit. Moreover, although reduced hepatic HuR expression correlates with MASLD severity [100], elevated HuR levels and cytoplasmic localization are associated with poor prognosis in both HCC [234] and heart failure [230], highlighting the need for context- and cell-specific therapeutic strategies in MASLD.

To address this challenge, several strategies have been proposed to enhance therapeutic specificity. One approach involves cell-targeted delivery, such as conjugating small-molecule inhibitors to retinol for selective uptake by retinol-storing HSCs, thereby limiting off-target effects while inhibiting fibrosis progression [235]. Additionally, rather than targeting HuR expression broadly, interventions could focus on specific functional aspects of HuR, including nuclear-to-cytoplasmic translocation, dimerization, post-translational modifications, or mRNA-binding activity—each of which may contribute differentially to HuR’s role in disease [236]. Importantly, genetic depletion of HuR in metabolic tissues such as the liver, heart, and adipose tissue typically does not impair basal tissue function unless these tissues are exposed to metabolic stressors such as lipid overload [42,119,226]. This observation suggests that therapeutic inhibition of HuR may exert selective toxicity under pathological conditions, providing an opportunity to titrate dosing and minimize adverse effects on healthy tissues. For example, KH-3, a small-molecule HuR inhibitor that blocks mRNA binding, has shown promise in selectively inhibiting HuR in tumor cells with high HuR expression, while sparing normal tissues [98]. A similar strategy could be employed to inhibit HSC activation during MASLD progression to cirrhosis and ultimately HCC, without compromising hepatocyte function.

While further research is needed to develop safe and effective HuR-targeted therapies, growing insight into HuR’s multifaceted role in metabolic disease presents promising opportunities for precision medicine. Unraveling the mechanisms by which HuR contributes to metabolic homeostasis and dysfunction may reveal novel therapeutic targets for MASLD and related cardiometabolic disorders. However, given HuR’s essential roles in maintaining cellular equilibrium across diverse tissues, caution is warranted in applying HuR inhibitors—particularly in the treatment of cancer or age- and inflammation-associated diseases.

## Figures and Tables

**Table 1.** Regulatory mechanisms governing HuR expression, subcellular localization and function.

Regulator	Context	Effect on HuR	Ref.

**Transcriptional**			
NFκB	Cell survival/proliferation in MKN74 cells	Long gene variant expression	[67]
SMAD	ATP depletion in LLC-PK1 cells	Short gene variant expression	[68]
**Post-transcriptional**			
HuR	Human diploid fibroblasts HeLa cells	Cytoplasmic localization	[36,69]
AUF1	HeLa cells	mRNA destabilization	[69,71]
miR-22	SW480 colorectal cancer cells	mRNA destabilization	[72]
miR-29	Acute myeloid leukemia cells	mRNA destabilization	[73]
miR-519	Human carcinoma cells	Impaired translation	[74]
**Post-translational**			
Phosphorylation			
CDK1	Irradiated HeLa cells	Nuclear localization	[55]
CHK2	H_2_O_2_-treated HeLa cells	Altered mRNA binding affinity	[74,77]
MAPK p38	Irradiated HaCaT human keratinocytes	Cytoplasmic localization	[39,79]
PKC-α	ATP-treated human mesangial cells	Cytoplasmic localization	[80]
PKC-δ	HCV-infected Huh7.5 cells	Cytoplasmic localization	[63]
Methylation			
CARM1	Human diploid fibroblasts	Altered mRNA binding affinity	[35]
Protein cleavage			
CASP3/7	Staurosporine-treated HeLa cells	Cytoplasmic localization; pro-apoptotic	[33,34]

**Table 2.** Regulation of mature mRNAs by HuR in both physiological and pathological conditions.

HuR Target mRNA ^1^	mRNA Function	HuR’s Effect on mRNA	Reference

*CCNB1*	Cell cycle progression	Stabilization	[30]
*CCND1*	Cell cycle progression	Stabilization	[40]
*CCNE1*	Cell cycle progression	Stabilization	[103]
*CDKN1A*	Cell cycle inhibition	Stabilization	[39]
*CSF2*	Pro-inflammatory	Stabilization	[88]
*FOS*	Rapid response gene	Stabilization	[22]
*FOXQ1*	Tumorigenesis	Stabilization	[98]
*NOS2*	Pro-inflammatory	Stabilization	[104]
*PTGS2*	Pro-inflammatory	Stabilization	[79]
*TGFB1*	Cell growth	Stabilization	[41]
*TNF*	Pro-inflammatory	Stabilization	[105]
*XIAP*	Anti-apoptotic	Enhanced Translation	[96]
*CDKN1B*	Cell cycle inhibition	Suppressed Translation	[97]

1 *CCNB1* (Cyclin B1), *CCND1* (Cyclin D1), *CCNE1* (Cyclin E1), *CDKN1A* (Cyclin dependent kinase inhibitor 1a), *CDKN1B* (Cyclin dependent kinase inhibitor 1b), *FOS* (FOS proto-oncogene), *CSF2* (Colony stimulating factor 2), *FOXQ1* (Forkhead box Q1), *NOS2* (inducible nitric oxide synthase), *PTGS2* (prostaglandin-endoperoxide synthase 2), *TGFB1* (Transforming growth factor β1), *TNF* (Tumor necrosis factor alpha), *XIAP* (X-linked inhibitor of apoptosis).

## Data Availability

Not applicable.

## Abbreviations

The following abbreviations are used in this manuscript:

ACAT: Acyl-CoA:cholesterol Acyltransferase
ACAT2: Alpha Smooth Muscle Actin
ADIPOQ: Adiponectin
AP-1: Activator Protein-1
APAP: Acetaminophen
APOB: Apolipoprotein B
APOE: Apolipoprotein E
APRIL: A Proliferation-Inducing Ligand
AMPK: AMP-activated Protein Kinase
ATGL: Adipose Triglyceride Lipase
BCL2: B-cell lymphoma 2
BECN1: Beclin-1
BMDMs: Bone Marrow-Derived Macrophages
CVD: Cardiovascular Disease
Casp9: Caspase-9
CCL2: Chemokine (C-C Motif) Ligand 2
CCNE1: Cyclin E1
CDK1: Cyclin dependent kinase 1
CDKN1A or p21: Cyclin dependent kinase inhibitor 1A
CEBPB: CCAAT enhancer binding protein β
c-FOS: Fos proto-oncogene, AP-1 transcription factor subunit
CHK2: Checkpoint kinase 2
CA: Cholic Acid
CDCA: Chenodeoxycholic Acid
COL1A1: Collagen 1a1
CREB: cAMP responsive element binding protein 1
CRM1: Chromosomal Region Maintenance 1
CYP: Cytochrome P450
CYTC: Cytochrome C
DAMP: Damage-Associated Molecular Patterns
DCA: Deoxycholic Acid
DLST: Dihydrolipoamide S-Succinyltransferase
DR5: Death Receptor 5
Elavl1: Embryonic lethal abnormal vision-like 1
ER: Endoplasmic Reticulum
ETS1: ETS proto-oncogene 1
FAO: Fatty Acid Oxidation
FFAs: Free Fatty Acids
GLP1RA: Glucagon-like peptide 1 receptor agonists
GM-CSF: Granulocyte-macrophage Colony-Stimulating Factor
HbA1c: Hemoglobin A1c
HCA: Hyocholic Acid
HCC: hepatocellular carcinoma
HEK293: Human Embryonic Kidney 293 Cells
HO-1/HMOX1: Hemeoxygenase-1
HMGCR: Hydroxymethylglutaryl-CoA reductase
HNS: HuR nucleocytoplasmic shuttling sequence
HSC: hepatic stellate cell
HuR: Human Antigen R; HuR
IL: interleukin
IL6: Interleukin 6
IL1B: Interleukin 1β
IMPα: Importin alpha
iNOS: Inducible Nitric Oxide Synthase
IL-6: Interleukin 6
IL-1 β: Interleukin 1β
I/R: Ischemia/reperfusion
JNK: c-JUN N-terminal Kinase
α-KGDH: alpha-ketoglutarate dehydrogenase
LC3: Atg8/microtubule-associated protein 1A/1B light chain
LCA: Lithocholic Acid
LncRNA: Long non-coding RNA
LPS: lipopolysaccharide
MASH: metabolic dysfunction-associated steatohepatitis
MASL: metabolic dysfunction-associated steatotic liver
MASLD: metabolic dysfunction-associated steatotic liver disease
MMP9: Matrix Metallopeptidase 9
MCD: Methionine Choline Deficient
MDM2: Mouse Double Minute 2 homolog
ω-MCA: ω-Muricholic Acid
NAFLD: Nonalcoholic Fatty Liver Disease
NDUFB6: NADH Ubiquinone Oxidoreductase subunit B6
NFE2L2: nuclear factor erythroid 2–related factor 2
NFκB: Nuclear Factor Kappa B
NKT: Natural Killer T Cells
NLRP3: NLR Family Pyrin Domain Containing 3
NOS2: Nitric Oxide Synthase 2
OPN: Osteopontin
p65: nuclear factor NF-kappa-B p65
PAMPs: Pathogen-associated Molecular Patterns
PDGF: Platelet-derived Growth Factor
PI3K/AKT: Phosphoinositide 3-kinase/AKT serine/threonine kinase 1
PNPLA2: Patatin-like Phospholipase Domain-containing 2
PPARG: peroxisome proliferator activated receptor gamma
pp32: phosphoprotein 32
PTEN: Phosphatase and Tensin Homolog
PTGS2 or COX2: prostaglandin-endoperoxide synthase 2
PTM: Post Translational Modification
PTMA: Prothymosin α
RBP: RNA binding protein
RMRP: RNA Component of mitochondrial RNA processing endoribonuclease
RNP-IP: ribonucleoprotein immunoprecipitation
ROS: Reactive Oxygen Species
SETα: SET nuclear proto-oncogene isoform α
SETβ: SET nuclear proto-oncogene isoform β
S1PR2: Sphingosine-1-phosphate Receptor 2
SIRT1: Sirtuin 1
α-SMA: Alpha Smooth Muscle Actin
SLC2A1: Solute Carrier Family 2 Member 1
SMAD: Mothers Against Decapentaplegic homolog
SQSTM-1/p62: Sequestosome 1
SREBP-2: Sterol Regulatory Element-Binding Protein 2
SOD2: Superoxide dismutase-2
SPHK1: Sphingosine Kinase 1
TCA: Taurocholic Acid
TCDCA: Tauro-chenodeoxycholic acid
T2DM: Type 2 Diabetes Mellitus
TGFB: Transforming Growth Factor Beta
TLR: Toll-like Receptor
TNF: Tumor Necrosis Factor
TRN1/2: Transportin 1 and 2
TTP: Tristetraprolin
TUDCA: Tauro-ursodeoxycholic Acid
UDCA: Ursodeoxycholic Acid
UQCRB: Ubiquinol–cytochrome c Reductase Binding Protein
UTR: Untranslated Region
VEGF: Vascular Endothelial Growth Factor
VLDL: Very-low Density Lipoprotein
